# Development of the gut microbiota in healthy children in the first ten years of life: associations with internalizing and externalizing behavior

**DOI:** 10.1080/19490976.2022.2038853

**Published:** 2022-02-19

**Authors:** Yangwenshan Ou, Clara Belzer, Hauke Smidt, Carolina de Weerth

**Affiliations:** a Department of Agrotechnology and Food Sciences, Laboratory of Microbiology, Wageningen University & Research, P.O. Box 8033, EH Wageningen, 6700 Netherlands; b Donders Institute for Brain, Cognition and Behaviour, Department of Cognitive Neuroscience, Radboud University Medical Center, P.O. Box 9010, 6500 GL Nijmegen, Netherlands

**Keywords:** infants, children, gut microbiota, development, internalizing behavior, externalizing behavior, *Prevotella*_9

## Abstract

**Background:**

Increasing evidence indicates that psychopathological disorders are associated with the gut microbiota. However, data are largely lacking from long-term longitudinal birth cohorts, especially those comprising low-risk healthy individuals. Therefore, this study aims to describe gut microbiota development in healthy children from birth till age 10 years, as well as to investigate potential associations with internalizing and externalizing behavior.

**Results:**

Fecal microbial composition of participants in an ongoing longitudinal study (*N* = 193) was analyzed at 1, 3 and 4 months, and 6 and 10 years of age by 16S ribosomal RNA gene sequencing. Based on these data, three clusters were identified in infancy, two of which were predominated by *Bifidobacterium*. In childhood, four clusters were observed, two of which increased in prevalence with age. One of the childhood clusters, similar to an enterotype, was highly enriched in genus-level taxon *Prevotella*_9. Breastfeeding had marked associations with microbiota composition up till age 10, implying an extended role in shaping gut microbial ecology. Microbial clusters were not associated with behavior. However, *Prevotella*_9 in childhood was positively related to mother-reported externalizing behavior at age 10; this was validated in child reports.

**Conclusions:**

This study validated previous findings on *Bifidobacterium*-enriched and -depleted clusters in infancy. Importantly, it also mapped continued development of gut microbiota in middle childhood. Novel associations between gut microbial composition in the first 10 years of life (especially *Prevotella*_9), and externalizing behavior at age 10 were found. Replications in other cohorts, as well as follow-up assessments, will help determine the significance of these findings.

## Introduction

The gut microbiota, mainly consisting of a vast number of bacteria,^1^ inhabits the gut of coelomate animals and has co-evolved with the hosts.^2^ These resident microorganisms play a crucial role in many aspects of health, including nutrition, immunity and neurophysiology.^3–5^ Evidence is accumulating that the gut microbiota also plays a crucial role in aspects of mental health and behavior.^5^ Therefore, maintaining normal diversity and function of the gut microbiota throughout development is essential for physical and mental health. The current study investigates gut microbial development from infancy to middle childhood, as well as potential relations of the gut microbiota with behavioral measures in healthy children.

In humans, infancy is commonly recognized as a critically important period for microbiota to colonize the gut.^6^ Before weaning, a healthy gut microbiota community is predominated by *Bifidobacterium*.^7^ Next to the *Bifidobacterium*-predominated type, researchers have identified several other infant gut bacterial types using cluster analyses. The identified clusters are characterized by *Bacteroides, Streptococcus, Enterobacteriaceae* or *Staphylococcaceae*,^8–10^ and are thought to develop as a result of complex extrinsic factors, such as child sex, birth weight, delivery mode, diet, and antibiotics.^11–19^ Changes in extrinsic factors can also lead individuals to transition between different clusters.^20^ While some studies suggested that by age 3, children have gut microbial profiles that strongly resemble those observed in adults,^21,22^ other cross-sectional studies concluded that an adult-like gut microbial ecosystem has not yet been completely established at this age.^23–25^ Compared to healthy adults, the fecal microbiota of healthy toddlers is characterized by a higher relative abundance of *Bifidobacterium*.^23^ Remarkably, *Bifidobacterium* remains more abundant in healthy school-aged children and older-aged adolescents, as compared to healthy adults.^24,25^ All of these cross-sectional studies imply that gut microbiota development may extend longer into childhood than previously thought, potentially due to long-term impacts of early extrinsic factors. However, information on healthy gut microbial development from longitudinal studies is largely lacking.

Researchers have gradually revealed a bidirectional communication between the gut microbiota and the host brain along the microbiota-gut-brain axis (MGBA), based on accumulating evidence from both animal and human studies mostly focused on clinical mental disorders, such as major depressive disorders and bipolar depression.^5,26–35^ In humans, childhood is regarded as a critical phase for behavioral problems to start emerging. Numerous studies showed that elevated childhood behavioral problems, such as internalizing (i.e., behavioral problems influencing children’s internal psychological environment, such as anxiety, depression, somatization, and social withdrawal symptoms) and externalizing problems (i.e., behavioral problems manifested in outward behavior such as antisocial behavior, aggression, hyperactivity, acting out, and hostility)^36^ are associated with higher chances of developing mental disorders and risky lifestyles in adulthood, which may in turn result in premature mortality.^37–40^ However, investigations about the MGBA in childhood are still at an early stage. Most of the existing studies are cross-sectional and mainly focused on children diagnosed with psychological disorders, such as autism spectrum disorder (ASD) and attention deficit/hyperactivity disorder (ADHD).^41–43^ Three previous longitudinal studies on community samples have reported associations of the gut microbiota with temperament, cognition, and behavioral problems in children until age 2 years.^44–46^ Two papers identified bacterial clusters associated with different behavioral patterns,^44,45^ while the third found increased internalizing problems in a *Prevotella*-depleted group.^46^ However, no studies have longitudinally investigated these links in healthy children beyond age 2.

The present study has two goals. First, we aimed to describe the normative development of the gut microbiota from birth till age 10 in a healthy community sample. To our knowledge, this has not been done before. We evaluated both short- and long-term associations of the gut microbial composition with extrinsic factors (i.e., birth weight, child sex, delivery mode, breastfeeding, and antibiotics) and determined whether the gut microbiota could be clustered into different successional patterns throughout the first 10 years on the basis of variance in microbial composition. Second, we aimed to investigate potential associations of the gut microbiota with internalizing and externalizing behavior in middle childhood in the same cohort. For this second aim, we raised three broad hypotheses based on scarce literature: internalizing and externalizing behavior would (1) differ between bacterial clusters; (2) explain general variance in microbial composition; (3) be related to alpha diversity or relative abundances of specific bacteria.

## Results

### First aim: gut microbiota development in the first 10 years of life

#### Gut bacterial clusters and transition patterns

**Figure 1.**
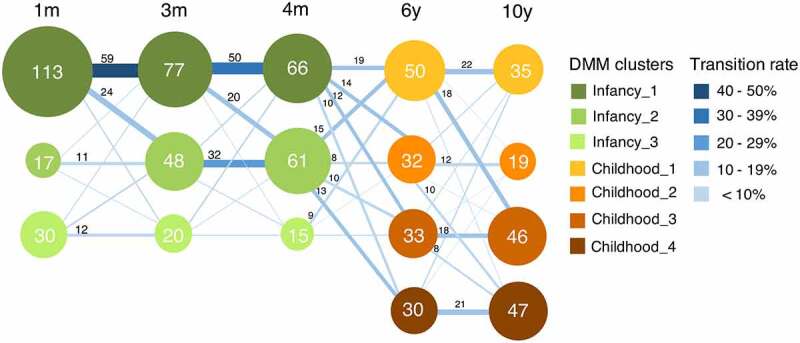
**Transition between bacterial clusters in the first 10 years** **of life.** Nodes represent clusters, with colors identifying a compositional cluster. Clusters were identified based on their compositional features at the genus level by Dirichlet multinomial mixtures (DMM) models. The size of the node indicates the number of individuals belonging to this cluster, which is displayed in the node. Lines are sized and colored based on the transition rate, with adjacent numbers representing the number of individuals transitioning from one cluster to another with increasing age. The numbers with transition rates below 6.0% are not shown.

To track gut microbiota development throughout infancy and childhood in the first 10 years of life, we stratified the participants into bacterial clusters based on their compositional features at the genus level by Dirichlet multinomial mixture (DMM) models. Based on microbial community composition, three bacterial clusters were obtained in infancy, and four clusters were found in childhood (Figure 1). While some individuals maintained the same bacterial composition over infancy or childhood, others transitioned to a different bacterial cluster when becoming older. At 1 month of age, 70.6% (113/160) of the infants belonged to Infancy_1, while fecal microbiota of 10.6% (17/160) and 18.8% (30/160) of the infants was classified as Infancy_2 and Infancy_3, respectively. Notably, the prevalence of Infancy_2 continuously increased with age throughout infancy, while the proportions of the other two clusters decreased. From infancy to childhood, no obvious transition pattern was observed. At age 6, Childhood_1 included 34.5% (50/145) of the children, while the other three clusters evenly shared the rest. From age 6 to 10, for individuals belonging to Childhood_1, 44.0% (22/50) remained in the same cluster, and another 36.0% (18/50) converted to Childhood_3. Similar transition patterns were also discerned in Childhood_2, of which 37.5% (12/32) of children remained in the same cluster, and another 31.3% (10/32) transitioned to Childhood_4. Meanwhile, 61.9% (39/63) of the children, belonging to either Childhood_3 or Childhood_4, stayed in the same clusters at age 10. As a consequence, 63.3% (93/147) of children ended up in Childhood_3 and Childhood_4 when reaching age 10.

#### Characteristics of gut bacterial clusters

**Figure 2.**
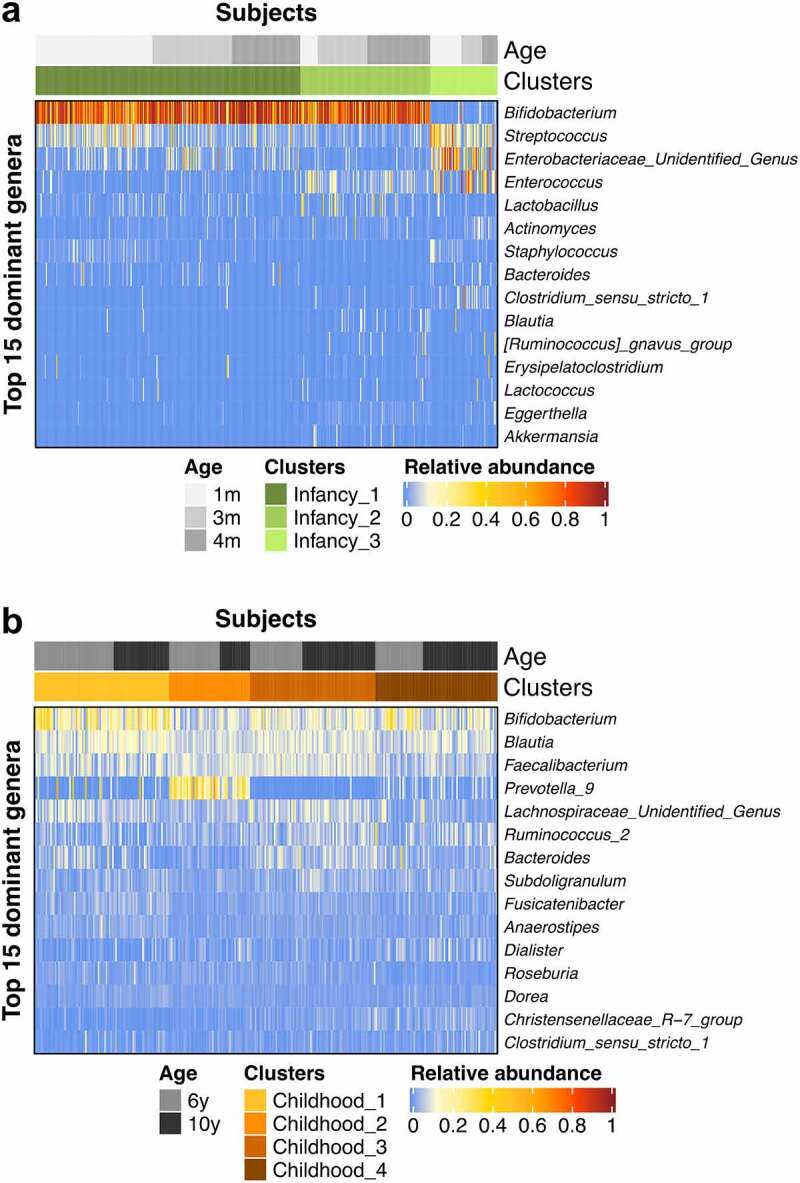
Heatmaps showing the relative abundances of the top 15 predominant genera in the bacterial clusters in infancy (a) and childhood (b).

Both Infancy_1 and Infancy_2 clusters were predominated by *Bifidobacterium*, and significantly differed in relative abundances of *Streptococcus*, an unidentified genus within the *Enterobacteriaceae* and *Enterococcus* (Figure 2a). Infancy_1 showed higher relative abundances of *Streptococcus* and an unidentified genus within the *Enterobacteriaceae*, and lower relative abundance of *Enterococcus*, in relation to Infancy_2. Compared to Infancy_1 and Infancy_2, Infancy_3 was depleted in *Bifidobacterium* but enriched in *Streptococcus, Enterococcus* and an unidentified genus from *Enterobacteriaceae*. In childhood, *Bifidobacterium* was among the most predominant genera, albeit at varying relative abundances (Figure 2b). In Childhood_1 *Bifidobacterium* was most predominant as compared to other genera, whereas Childhood_2 was predominated by *Prevotella*_9 at an average relative abundance of 24.5 ± 14.4%, which was much higher than in other clusters (Chilhood_1, 4.1 ± 11.4%; Childhood_3, 0.1 ± 1.0%; Childhood_4, 3.3 ± 4.4%).

To further describe the features of bacterial clusters, we compared the phylogenetic alpha diversity and beta diversity between them (Figures S1 and S2). Significant differences in alpha diversity indices between bacterial clusters reflected the results of DMM clustering in the current study.

To describe the potential functional differences between bacterial clusters, we exploratorily applied the Picrust2 (phylogenetic investigation of communities by reconstruction of unobserved states) method,^47,48^ based on 16S rRNA gene sequence data. In total, 2651 KEGG orthologs and 288 MetaCyc pathways were obtained over the study period. In infancy, 14 KEGG orthologs with average relative abundances more than 0.5% were significantly different between bacterial clusters after FDR adjustment (Table S20), while 13 KEGG orthologs differed significantly in childhood (Table S21). We found the function beta-galactosidase was predicted to be decreased in bacterial cluster Infancy_3 (0.19 ± 0.16%) compared with Infancy_1 and Infancy_2 (0.68 ± 0.17 and 0.67 ± 0.19%). In later life, beta-glucosidase was observed significantly increased in Childhood_2 (0.84 ± 0.17%) as compared to the other three childhood bacterial clusters (0.67 ± 0.16, 0.62 ± 0.07, and 0.6 ± 0.11%). Regarding MetaCyc pathways with average relative abundances more than 0.5%, 72 of them were significantly different after correction in infancy (Table S22), and 88 differed significantly in childhood (Table S23). These MetaCyc pathways mainly covered degradation and biosynthesis of carbohydrates and amino acids. In the first several months, pathways of *Bifidobacterium* shunt, mixed acid fermentation, L-arginine biosynthesis I and II, and superpathway of aromatic amino acid biosynthesis were significantly reduced in Infancy_3 as compared to Infancy_1 and Infancy_2. Pathways of L-arginine biosynthesis I and II were observed significantly depleted in Childhood_2 as compared to the other three bacterial clusters in childhood.

In addition to microbial compositional features, we also exploratorily investigated whether bacterial clusters differed on population characteristics, namely delivery mode, child sex, breastfeeding, medications, diseases, etc., in infancy and childhood (Tables S1 and S2). Delivery mode was significantly different between infant bacterial clusters, of which Infancy_3 showed highest rates in C-section and assisted vaginal delivery. Food frequency was also compared between bacterial clusters in childhood, and no significant differences were observed (Table S3).

#### Associations of gut microbial composition with extrinsic factors

To determine to what extent extrinsic factors (i.e., birth weight, child sex, delivery mode, breastfeeding, and antibiotics) can explain the observed variation in microbiota composition, their simple effects (i.e., the impact of one factor on gut microbiota without taking other factors into account) were measured separately per time point, as well as for all infancy and childhood samples, respectively (Tables S4 and S5). In infancy, breastfeeding significantly explained 1.0%, 1.4% and 1.2% of adjusted variances without the biases in microbial composition, in separate analyses at age 1, 3 and 4 months. None of the other factors tested, i.e., delivery mode, birth weight and child sex, significantly contributed to explaining the observed variation in microbial composition, in separate analyses at infancy time points. In childhood, no significant simple effects were observed at single time points.

In analyses pooling all infancy samples together, child age was found having the most predominant significant effect (1.0%), followed by breastfeeding and delivery mode (0.7% and 0.2%). With respect to simple effects of extrinsic factors in childhood, breastfeeding significantly explained around 0.3% of adjusted variance in microbial composition in the pooled data of ages 6 and 10. Similar to infancy, in the period from age 6 to 10, child age significantly explained the most observed variance in microbial composition (0.9%) as compared to other extrinsic factors of which only breastfeeding explained significant variance (0.3%).

Next to it, we measured conditional effects (i.e., the impact of individual factors when partitioning out effects from other factors) of the significant extrinsic factors obtained from pooled data (Table 1). These extrinsic factors included (1) Child age, delivery mode, and breastfeeding for infancy and (2) Child age and breastfeeding for childhood. After partitioning out total explained variance, selected infancy and childhood extrinsic factors were still able to significantly explain partial variance.

**Table 1.** Conditional effects of extrinsic factors with significant simple effects on gut microbiota in infancy and childhood

	R^2^%	Adjusted R^2^%	*p* Value	VIF	Number of individuals	Number of genera
**1–4 months**						
Age	1.051	0.817	0.001*	1.017	411	155
Delivery mode	0.639	0.16	0.001*	1.724 (CS);		
				1.723 (NAVD)		
Breastfeeding	0.791	0.556	0.001*	1.017		
**6–10 y**						
Age	1.554	0.869	0.001*	1.001	144	181
Breastfeeding	1.017	0.329	0.010*	1.001		

Significance was determined based on 1000 permutations. Asterisks indicate *p* value < 0.05. VIF: variance inflation factor. CS: C-section. NAVD: non-assisted vaginal delivery.

**Figure 3.**
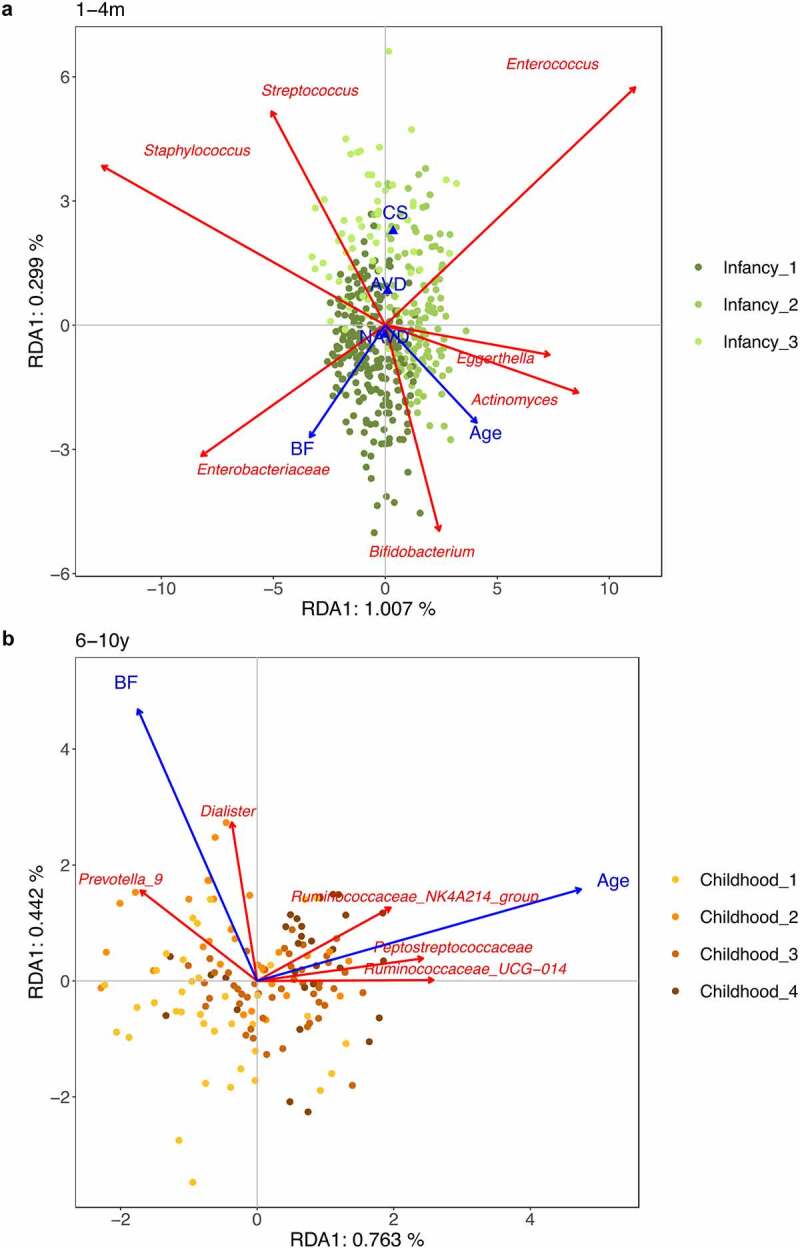
**RDA plots of extrinsic factors with significant conditional effects on gut microbial composition.** (a) RDA plot based on microbiota profiles at the age of 1, 3 and 4 months. (b) RDA plot based on microbiota profiles at the age of 6 and 10 years. RDA plots are displayed based on Bray-Curtis distance matrices computed from log-transformed data at the genus level. Unidentified genera are shown at the family level. To clarify, we displayed adjusted variances along axes, which were corrected to be without biases. Generally, the value of adjusted variances is less than that of original variances. CS: C-section. AVD: assisted vaginal delivery. NAVD: non-assisted vaginal delivery. BP: breastfeeding proportion.

To gain more insights into the associations between the gut microbiota and extrinsic factors with significant conditional effects, we performed analyses on the pooled infancy data and the pooled childhood data (Figure 3). During infancy, breastfeeding was positively associated with increased relative abundances of *Bifidobacterium* and an unidentified genus within *Enterobacteriaceae*. Infants with C-section showed higher levels of *Streptococcus* and *Enterococcus*, and lower levels of *Bifidobacterium*. With increasing age from 1 to 4 months, *Bifidobacterium, Actinomyces* and *Eggerthella* also increased in relative abundances. Over childhood, age was positively related to higher relative abundances of unidentified genera from the *Ruminococcaceae* and *Peptostreptococcaceae*. In the same age period, higher breastfeeding was associated with higher relative abundances of *Prevotella*_9 and *Dialister*.

### Second aim: associations of the gut microbiota with internalizing and externalizing behavioral measures in middle childhood

#### Bacterial clusters and behavior

We did not find any significant associations between bacterial clusters and child internalizing and externalizing behavior (Figures S3 and S4).

#### RDA models

Before exploring associations between the gut microbiota and behavioral measures, we first assessed which behavioral measures were capable of significantly explaining variance in microbial composition with and without accounting for the extrinsic factors studied in the first aim, by using Redundancy Analysis (RDA) models. Without accounting for these factors, internalizing behavior, evaluated by the Strengths and Difficulties Questionnaire (SDQ) maternal reports at 10 years of age, was able to explain the variance in microbial composition at 1 month of age (*p* = 0.050; Table S6).

As for samples in childhood (Table S7), maternal-reported externalizing behavior, measured by the SDQ at age 10, significantly explained variance in gut microbial composition at age 6. Remarkably, we found both internalizing and externalizing behavior, assessed by the maternal SDQ at age 10, were able to significantly explain variation in the gut microbiota in the period from age 6 to 10. When taking also significant extrinsic factors into account, i.e., child age and breastfeeding (Table 2), we found that the maternal SDQ reports of externalizing behavior at age 10 still significantly explained variance in microbial composition during childhood. However, internalizing behavior was no longer significant in this model.

**Table 2.** Partial variance in microbial composition in childhood explained by behavioral problems at age 10 as reported by the mother (SDQ)

	Behavior	R^2^%	Adjusted R^2^%	*p* Value	VIF	Number of genera
**6–10 y**						
SDQ_M_10y	Internalizing	0.851	0.127	0.148	1.062	181
	Externalizing	1.098	0.379	0.005*	1.058	

Significance was determined based on 1000 permutations. Asterisk indicates *p* value < 0.05. VIF: variance inflation factor. CS: C-section. NAVD: non-assisted vaginal delivery.

**Figure 4.**
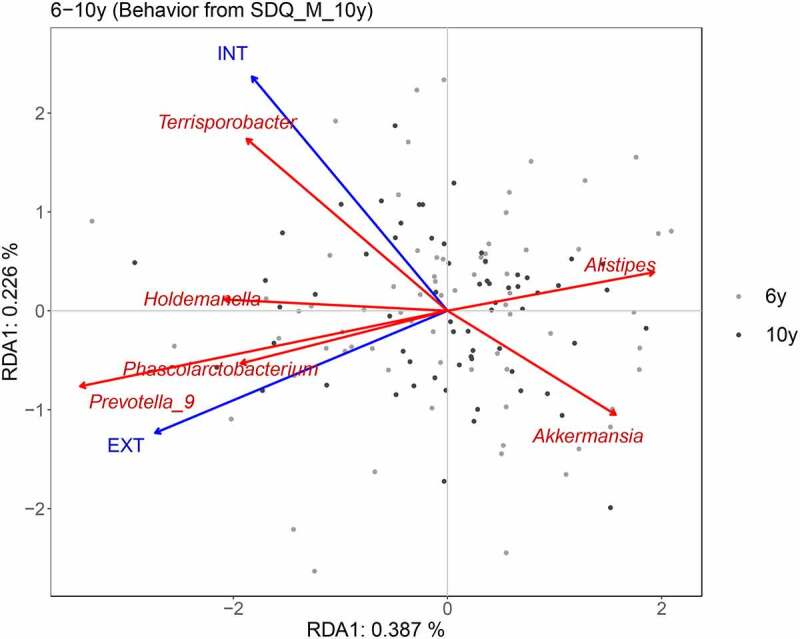
**Partial RDA plot indicating associations of genera in childhood with internalizing and externalizing behavior at age 10 as reported by the mother (SDQ).** RDA plots are displayed based on Bray-Curtis distance matrices calculated from log-transformed data at the genus level. Child age and breastfeeding were accounted for. To clarify, we displayed adjusted variances along axes, which were corrected to be without biases. Generally, the value of the adjusted variance was less than that of the original variance. INT: internalizing behavior. EXT: externalizing behavior.

To specifically explain the associations of pooled gut microbiota of ages 6 and 10 with internalizing and externalizing behavior assessed by the maternal SDQ at age 10, partial RDA was performed by accounting for age and breastfeeding (Figure 4). Externalizing behavior showed positive associations with relative abundances of *Prevotella*_9 and *Phascolarctobacterium*. In addition, more internalizing behavior was related to reduced relative abundance of *Akkermansia*, and more externalizing behavior was associated with decreased relative abundance of *Alistipes*. Finally, a higher relative abundance of *Terrisporobacter* was found in individuals with higher internalizing behavior scores.

#### PRC models

**Figure 5.**
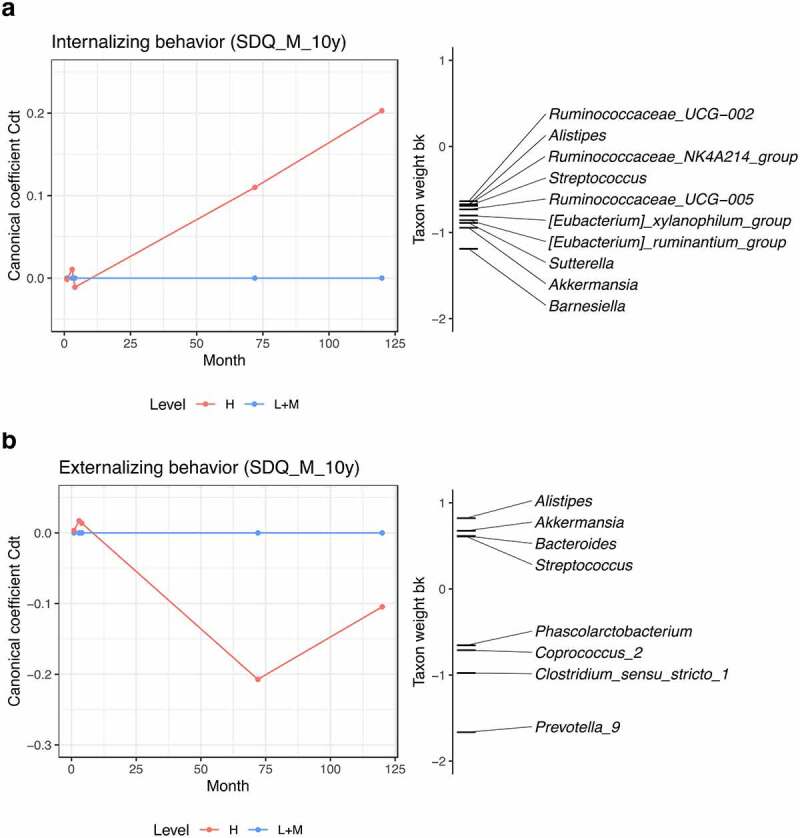
**PRC analysis of internalizing (a) and externalizing behavior (b) at age 10 as reported by the mother (SDQ).** Behavior groups are set based on quartiles. H level includes individuals with scores in the top quartile, and L+M level includes the bottom three quartiles. L+M level is used as reference (Low+Medium; baseline). Canonical coefficients indicate the differences between H and L+M. The wider the distance between H and L+M, the more dissimilar they are to each other. The taxon weight reflects for which taxa the compositional variation is best represented by the PRC model. The direction of abundance change is determined jointly by the signs of canonical coefficient and taxon weight. Same signs indicate increased relative abundance, while reverse signs represent reduced relative abundance. Genera with absolute values of taxon weights less than 0.60 are not displayed. Genera data was pre-processed with log-transformation.

In order to assess emerging associations of gut microbiota composition as measured during early infancy and childhood with internalizing and externalizing behavior at age 10, we performed Principal Response Curves (PRC) analyses (Figure 5). This method can be used to assess temporal trajectories of dissimilarity between behavior groups with different scores, and to select genera with relatively large changes in relative abundances across the first 10 years of life. With respect to internalizing behavior, measured by the maternal SDQ at age 10, no obvious differences were observed between high (H) and low/medium (L + M) score groups in the first 4 months of life, whereas the dissimilarity in microbial composition between groups started changing somewhere between 4 months and 6 years.

Similarly, with respect to externalizing behavior, measured by the same questionnaire at the same age, differences between behavior groups started emerging between 4 months and 6 years. Among the time points included in this study, the difference between groups of externalizing behavior was highest at age 6, and then decreased again at age 10. Children belonging to the top quartile (H), tended to show higher levels of *Prevotella*_9 and *Clostridium*_sensu_stricto_1, and reduced relative abundances of the genera *Alistipes, Akkermansia, Bacteroides* and *Streptococcus* (Figure 5). As the coefficients for group H are negative compared to the baseline, negative taxon weights indicate positive correlations.

Furthermore, we compared the differences in relative abundances of the genera selected from PRCs between behavior groups at each age. With respect to internalizing behavior, the genera *Akkermansia, Alistipes, Sutterella, Barnesiella*, and two genus-level groups of *Eubacterium* were observed reduced in individuals belonging to the H group at age 10, although not significantly after FDR adjustment (Table S8). As for externalizing behavior, *Alistipes* was depleted, and *Phascolarctobacterium, Coprococcus*_2, *Clostridium*_sensu_stricto_1 and *Prevotella*_9 were increased in relative abundances in the H group at age 6 (Table S9). After FDR correction, *Clostridium*_sensu_stricto_1 and *Prevotella*_9 were observed with *p* values less than 0.10. At age 10, the relative abundance of *Clostridium*_sensu_stricto_1 remained higher in H group individuals, albeit insignificant after FDR correction.

#### MLM models

Multilevel models (MLM) were used due to their ability in processing time-series data. These models included the following extrinsic factors: child age, delivery mode, breastfeeding, birth weight, and child sex in infancy, and the same factors, as well as age of solid food introduction and antibiotic treatments, in childhood. For the maternal SDQ at age 10, we found that internalizing behavior was moderately positively related to phylogenetic alpha diversity during infancy (Table 3), while externalizing behavior was not related to alpha diversity or relative abundances of specific genera in infancy. In childhood, *Alistipes* was found to be significantly negatively associated with externalizing behavior assessed by the maternal SDQ at age 10. In addition, higher relative abundances of *Prevotella*_9 and *Phascolarctobacterium* were significantly associated with increased externalizing behavior. Interestingly, similar associations of *Prevotella*_9 and *Phascolarctobacterium* with externalizing behavior were further validated in the SDQ child reports at age 10 (Table S10). MLM models were also conducted for behavior assessed by the maternal CBCL at age 6, however, no consistent associations were found (Table S11).

**Table 3.** MLM models for internalizing and externalizing behavior at age 10 as reported by the mother (SDQ)

	SDQ_M_10y_Internalizing			SDQ_M_10y_Externalizing		
Response variable	Estimate	95% CI	*p* Value	Estimate	95% CI	*p* Value
**1–4 months**						
Phylogenetic diversity	0.127	[−0.014, 0.269]	0.088	−0.052	[−0.189, 0.086]	0.473
*Streptococcus*	0.08	[−0.075, 0.234]	0.327	−0.081	[−0.232, 0.071]	0.31
*Clostridium_sensu_stricto_1*	−0.047	[−0.232, 0.138]	0.628	0	[−0.179, 0.180]	0.999
**6–10 y**						
Phylogenetic diversity	−0.054	[−0.248, 0.139]	0.61	0.084	[−0.110, 0.278]	0.426
*Streptococcus*	0.166	[−0.078, 0.407]	0.21	−0.192	[−0.436, 0.047]	0.147
*Clostridium_sensu_stricto_1*	0.18	[−0.032, 0.393]	0.122	0.186	[−0.025, 0.397]	0.107
*Bacteroides*	0.089	[−0.077, 0.253]	0.323	−0.172	[−0.338, −0.008]	0.058
*Barnesiella*	−0.181	[−0.440, 0.079]	0.2	−0.143	[−0.402, 0.114]	0.309
*Prevotella_9*	0.222	[−0.194, 0.637]	0.326	0.614	[0.192, 1.035]	0.009*
*Alistipes*	−0.088	[−0.319, 0.148]	0.489	−0.333	[−0.566, −0.101]	0.010*
*Coprococcus_2*	−0.024	[−0.289, 0.231]	0.863	0.172	[−0.083, 0.427]	0.219
*Ruminococcaceae_NK4A214_group*	0.034	[−0.184, 0.250]	0.774	−0.145	[−0.355, 0.072]	0.212
*Phascolarctobacterium*	0.065	[−0.224, 0.355]	0.677	0.339	[0.046, 0.632]	0.036*
*Sutterella*	−0.117	[−0.365, 0.124]	0.378	0.101	[−0.145, 0.341]	0.448
*Akkermansia*	−0.254	[−0.537, 0.033]	0.103	−0.158	[−0.439, 0.123]	0.304
*[Eubacterium]_ruminantium_group*	−0.097	[−0.321, 0.128]	0.428	0.046	[−0.174, 0.270]	0.703
*[Eubacterium]_xylanophilum_group*	−0.21	[−0.415, −0.005]	0.063	0.071	[−0.131, 0.271]	0.52
*Ruminococcaceae_UCG-002*	−0.095	[−0.303, 0.107]	0.396	−0.056	[−0.257, 0.149]	0.615
*Ruminococcaceae_UCG-005*	−0.077	[−0.349, 0.199]	0.606	−0.116	[−0.389, 0.158]	0.435

Numbers of individuals included in MLM models are listed in Table S12. Genera with prevalence less than 0.20 were not included in MLM models. Detailed information of genera prevalence can be reached in Table S13. Covariates used to be accounted for and their GVIFs were summarized in Tables S14 and S15. Asterisk indicates *p* value < 0.05.

## Discussion

In our study, three distinct bacterial clusters were identified in samples taken during the first 4 months of life. This is in line with previous studies focusing on the first half year of life,^8–10^ both for the number of clusters and the bacterial cluster composition. Regarding Infancy_1 and Infancy_2, they were predominated by *Bifidobacterium* with numerical predominance values at 77.5 ± 20.9% and 75.2 ± 20.4%, which are similar to the numerical predominance values of *Bifidobacterium*-predominated clusters in the three previous studies.^8–10^ Contrary to the *Bifidobacterium*-enriched clusters, Infancy_3 was depleted in *Bifidobacterium* but enriched in *Streptococcus* and an unidentified genus within *Enterobacteriaceae*; these characteristics were also found in previously reported clusters.^8–10^ Notably, the identification of these *Bifidobacterium*-depleted clusters varies between studies. For instance, Dogra *et al*. found two, rather than one, *Bifidobacterium*-depleted clusters, enriched in *Streptococcus* and *Enterobacteriaceae*, respectively.^10^ This may be due to the differences in clustering methods and study populations. Also, Borewicz *et al*. described a *Bacteroides*-predominated cluster that was absent in our study.^8^ High intra-individual variability of the gut microbiota may explain these different findings, since we used the same clustering method and included participants from the same country as Borewicz *et al*. Future large-scale studies and meta-analyses may help clarify these clustering issues in infant populations.

Remarkably, the infant cluster transition patterns in our study were highly similar to those previously reported by other studies.^10,49^ The prevalence of *Bifidobacterium*-enriched clusters was increased from 1 to 4 months of age, while the ratio of Infancy_3 was reduced. Infancy_3 was identified with the highest proportion of C-section and assisted vaginal deliveries, while no differences in breastfeeding were observed between infant clusters in our study. The shift toward a *Bifidobacterium*-enriched microbial community in infants from the Infancy_3 cluster may imply a quick adaption to environmental changes, such as the initiation of breastfeeding. Given the similarity between Infancy_1 and Infancy_2, generating more specific profiles of *Bifidobacterium* species and strains may help enhance their differentiation. In sum, our study provides further support on the consistency of gut bacterial clusters in infancy, regardless of differences between studies in sample size, collection period and clustering method.

In fecal samples taken at 6 and 10 years of age, we distinguished four bacterial clusters and delineated how children transitioned between these clusters, with two clusters separately predominated by *Bifidobacterium* and *Prevotella*_9 and the other two enriched in *Bifidobacterium, Blautia* and *Faecalibacterium*. Our clusters showed similarities and differences to those of a recent study by Zhong *et al*. in healthy Dutch school-aged children (mean age 7.3 years, ranging from 6 to 9).^50^ Our Childhood_1 closely resembled the *Bifidobacterium*-dominated cluster reported by Zhong *et al*. and showed a similar relative abundance of *Bifidobacterium* at 21.6 ± 12.0%. In adults, the relative abundance of *Bifidobacterium* normally ranges from 2% to 14%.^51^ The other three childhood clusters observed in our study were within this range. Although a *Bifidobacterium*-predominated microbiota is commonly known to be beneficial for infants, this type of cluster may lack maturity in children and adults.^20^ Moreover, Childhood_1 displayed the lowest diversity among all four clusters (Fig. S1; also reported by Zhong *et al*.). This finding further supports the notion of immaturity of Childhood_1, as the lower diversity may be paired to a corresponding lower functional potential that may not fully meet the requirements of complex carbohydrate utilization and butyrate production in later life.^50^ Childhood_2 was similar to a *Prevotella*-predominated cluster observed by Zhong *et al*., and also to one of the three human adult enterotypes.^52^ These microbial community types exhibit approximately 20% of *Prevotella*, a genus positively associated with carbohydrate intake and fiber consumption.^53,54^ In contrast to the findings of Zhong *et al*., in our study, no *Bacteroides*-predominated cluster was found. Although Childhood_3 showed the highest level of *Bacteroides* across our clusters, the relative abundance of this genus (6.6 ± 4.4%) was lower than in the corresponding community type reported by Zhong *et al*. (near 20%). Childhood_3 was enriched in a group of near evenly distributed genera, including *Bifidobacterium, Blautia, Faecalibacterium*, and an unidentified genus within *Lachnospiraceae* (12.3 ± 6.2%, 10.2 ± 3.1%, 10.0 ± 3.5% and 8.49 ± 5.6%). Childhood_4 had similar levels of *Bifidobacterium* and *Blautia* (13.3 ± 10.2% and 8.9 ± 4.2%) as Childhood_3, while comprising lower levels of *Faecalibacterium* and an unidentified genus within *Lachnospiraceae* (5.9 ± 3.2% and 3.7 ± 4.4%) than Childhood_3. Both Childhood_3 and Childhood_4 showed more diverse and more evenly distributed microbiota than *Bifidobacterium*-predominated Childhood_1 and *Prevotella*_9-predominated Childhood_2; these features may allow more complex functions in Childhood_3 and Childhood_4, and hence may mark a mature gut microbiota community for children in middle childhood or at a later age.

The differences between the studies may be attributed to age, as Zhong *et al*. included consecutive time points from age 6 to 9, while the present study was specifically focused on ages 6 and 10. This particular period, spanning 4 years and reaching into early puberty, may be of relevance for gut microbial development. Indeed, from age 6 to 10, we observed an overall progressive transition of children from Childhood_1 and Childhood_2 to Childhood_3 and Childhood_4, both displaying higher alpha diversity than the other two clusters, hinting at a trend toward increasing microbial functional capacity from age 6 to 10. This also indicates that in healthy children gut microbial development appears to continue at least until early puberty. Though diet is regarded as an important factor influencing the gut microbiota, we did not find differences between childhood bacterial clusters with respect to the children’s dietary intake. Note, however, that this may be due to the fact that we only measured food frequency at age 10, while the gut bacteria were assessed at ages 6 and 10.

We further investigated potential functional differences of the gut microbiota between bacterial clusters in an exploratory manner by using the Picrust2 approach. In general, we noticed that multiple predicted metabolic functions (i.e., KEGG orthologs and MetaCyc pathways) varied between bacterial clusters in infancy and childhood. For example, in infancy, we observed that the level of KEGG ortholog beta-galactosidase, an enzyme catalyzing the hydrolysis of beta-galactosides like lactose, was lower in Infancy_3 in comparison with the other two infant bacterial clusters. Beta-galactosidase has been found prevalent in *Bifidobacterium* species.^55^ Consistent with this, Infancy_3 showed the lowest level of *Bifidobacterium*; hence, the depletion of *Bifidobacterium* may explain the reduction of beta-galactosidase in Infancy_3. In childhood, we found that the relative abundance of the KEGG ortholog beta-glucosidase, an enzyme hydrolysing various glycosides like cellulose coming from plant foods, was highest in bacterial cluster Childhood_2. Childhood_2 was enriched in a fiber-favoring bacterium *Prevotella*_9. As a consequence, this cluster can be considered to have a higher ability of utilizing cellulose, which is in line with our finding. As for differences in MetaCyc pathways, we observed that the biosynthesis of precursors (i.e., aromatic amino acids) for neurotransmitters (i.e., serotonin, dopamine and norepinephrine), was decreased in Infancy_3. This bacterial cluster also showed decreases in mixed acid fermentation and *Bifidobacterium* shunt, which might indicate a reduction in short-chain fatty acids (SCFAs) production. Although the role of SCFAs on the MGBA has not been clearly elucidated, they are speculated to have considerable impacts.^56^ In both Infancy_3 and Childhood_2, we noticed decreased levels in predicted functions of L-arginine biosynthesis I and II. L-arginine supplementation has been reported to stimulate glutamate decarboxylation in *Lactococcus lactis*, which in turn increases the production of the neurotransmitter gamma-aminobutyric acid (GABA).^57^ However, it is unknown if other bacteria have similar interactions of L-arginine with GABA. Finally, note that there are two main limitations of any function prediction tool based on marker genes such as Picrust2.^47^ The first is the bias caused by the reference database, and the second is that the resolution cannot distinguish strain-specific functionality. Hence, these findings of predicted functions must be seen as exploratory and interpreted with caution.

Regarding extrinsic factors, breastfeeding was found to explain a moderate amount of variance in infant gut microbial composition, similarly to our previous findings in which breastfeeding explained 2–6% of the variance.^58^ In line with previous studies,^18,21,59^ increased breastfeeding was found related to higher levels of *Bifidobacterium* in the first 4 months of life. Surprisingly, early-life breastfeeding was also associated with the gut microbiota in the period from 6 to 10 years of age. This finding tied well with observations by Zhong *et al*., who uncovered a persistent effect of breastfeeding duration on the gut microbiota based on community samples at school age.^50^ Although it is widely accepted that breastfeeding only prominently affects the gut microbiota in infancy or early childhood,^18,60^ both Zhong’s and our findings may indicate an extended influence of breastfeeding on shaping microbial composition and even function. In addition, we found that breastfeeding was positively associated with increased *Prevotella*_9 in childhood. *Prevotella*, as a genus prevalent in populations consuming fiber,^53^ has been found tightly linked to glucose metabolism.^61^ However, in this study, it is unknown if the increased level of *Prevotella*_9 is caused by breastfeeding or other relevant dietary factors. In a recent study based on another population, we found that longer exclusive breastfeeding duration was associated with a healthier child diet at age 3 years,^62^ note though that, as mentioned before, diet at age 10 was unrelated to child gut microbiota. Further studies aiming to validate this association and explore causality are hence needed to clarify these issues. With respect to antibiotic use, due to the fact that very few infants were treated with antibiotics in our study, we did not take early antibiotic use into consideration. Note, however, that in populations where antibiotic use in infants is commonplace, antibiotic treatments should be included as a potential confounder as there is evidence that they not only affect the gut microbial composition but might also affect later neurodevelopment.^63–65^

Regarding associations between the gut microbiota and child behavior, we found no associations of the bacterial clusters with internalizing and externalizing behavior measured by maternal and child reports at age 6 and 10. In earlier studies, clustering methods were also adopted with the aim of exploring links of the child gut microbiota with subsequent temperament at 6 months and cognition at 2 years.^44,45^ Compared to these studies in which the microbial composition was analyzed at one selected time point, in the present study we used five time points in the first 10 years of life to more comprehensively delineate relations between the bacterial clusters and problem behavior. Although we did not find that bacterial clusters were related to problem behavior in our study, this does not imply that clustering methods were inappropriate to use. Indeed, clustering methods are highly suitable for high-dimensional data. Also, it is worth noting that there can be a moderate relation between the gut microbiota and problem behavior, the substantiation of which might require larger datasets to reflect this relation. Furthermore, variation in microbial composition does not directly provide information about differences in microbial function involved in MGBA. In other words, different microbial communities may hold similar gene potential. Limited by the 16S rRNA sequencing technique, we were only able to explore function with the Picrust2 method in the current data. This method has shortcomings that can be avoided by using metagenomics in combination with metabolomics analyses in future studies.

With respect to specific bacteria, based on several complementary statistical methods, including RDA, PRC and MLM models, we found that the relative abundances of *Prevotella*_9 and *Phascolarctobacterium* in samples taken at age 6 to 10 were positively associated with increased mother-reported externalizing behavior at age 10, while a negative association was observed in the level of *Alistipes* with the same externalizing behavior at the same age. A previous longitudinal study in toddlers found that a higher relative abundance of *Prevotella* at 1 year of age was related to more internalizing behavior, but not externalizing behavior, at age 2.^46^ The large age gap and different assessment moments may explain the differences between the two studies. Comparing our findings with studies focusing on children with psychopathology, we find that autistic children (4 to 11 years), commonly exhibiting co-occurring externalizing problems,^66^ showed increased abundances of microbial groups including *Prevotella, Bacteroides* and *Porphyromonas*, compared to healthy controls.^67^ In contrast, another study found that *Prevotella* was reduced in children with autism (3 to 16 years).^68^ Apart from autism, ADHD has also been shown to be associated with externalizing behavior in adolescence.^69^ For children with ADHD, two previous studies reported no changes in *Prevotella* abundance,^43,70^ whereas Kristensen *et al*. found decreased levels of *Prevotellaceae*.^71^ In sum, there is no well-defined link between *Prevotella* and behavioral problems and mental disorders, just as at the physical health level, *Prevotella* has been related to the consumption of beneficial plant-rich diets, but also to harmful chronic inflammation.^72^ As a large genus, *Prevotella* includes around 40 different species that greatly vary in their genetic potential.^72^ In this case, using metagenomic analyses to characterize the *Prevotella* population at higher taxonomic resolution, i.e., species or strain level, would be helpful to better understand a more specific potential interaction with host behavior. With respect to *Phascolarctobacterium*, a systematic review showed its relative abundance was higher in patients with major depressive disorder (MDD) than controls,^73^ while Li *et al*. reported that it was positively related to improved mood in adults with the same dietary structure.^74^ Though these studies reflect that *Phascolarctobacterium* is related to internalization-relevant mental problems, it is worth noting that internalizing and externalizing behavior can co-exist in children and may lead to opposite behavioral problems at a later age.^75–77^ As for *Alistipes*, earlier studies found its role was divergent in MDD and ASD.^41,67,73^ As described earlier, distinct behavioral issues can co-occur and even predict the opposite one in the same child; this does not only work for MDD but also for ASD which is often accompanied by greater aggression.^78^ Given the complexity of mental problems, the associations of *Phascolarctobacterium* and *Alistipes* with externalizing behavior need to be interpreted with caution.

Strengths of this study include the prospective, lengthy longitudinal design with repeated gut microbial sampling in healthy community children. Additionally, behavioral measures were reported by both mothers and children and at two different ages, and a series of sophisticated and complementary statistical analyses were performed. A limitation of the study is the restricted taxonomic resolution of the 16S rRNA gene sequence data used in this study, which does not permit us to distinguish bacteria at the species or strain level.

In sum, in this study we identified three bacterial clusters in infancy and four in childhood and explored transitional trajectories of individuals through these clusters in the first 10 years of life. These clusters exhibited similarities as well as differences to previously identified clusters. Among the different extrinsic factors studied, breastfeeding stood out by having marked associations with the gut microbiota up till age 10, implying an extended role in shaping gut microbial ecology. With respect to problem behavior, high relative abundances of *Prevotella*_9 and *Phascolarctobacterium* and a low level of *Alistipes* in middle childhood were associated with increased externalizing behavior at age 10. In the future, strain-resolved metagenomic sequencing, as well as specific sets of qPCR assays, can provide a better understanding of the potential role of *Prevotella*_9 in child behavior. Additionally, determining behaviorally relevant fecal metabolites will help bridge the gap between association and causality. Finally, to take a step further in understanding the development of the gut microbiota throughout childhood, as well as its relations with child behavioral phenotypes, healthy longitudinal cohorts with a higher frequency of gut microbial sampling (e.g., yearly samples throughout childhood) are direly needed.

## Materials and methods

### Participants

Participants were identified from an ongoing longitudinal Dutch study named BIBO (Basale Invloeden op de Baby Ontwikkeling),^79^ consisting of healthy low-risk individuals (N = 193), with approval from the ethical committee of the Faculty of Social Sciences of the Radboud University (ECG300107, ECG13012012, SW2017-1303-497 and SW2017-1303-498).

### Data collection procedures

Parents were instructed to collect fecal samples in sterilized plastic tubes by using the scoop attached to the tube cap, when their children were 1, 3 and 4 months of age, and 6 and 10 years of age. Infancy samples were collected from diapers, and childhood samples were collected immediately after defecation from potties or toilets without contact with the toilet water. The tubes were then placed in clean plastic bags provided by investigators before being temporarily kept in the freezer (−20°C). Samples were transported to the Laboratory of Microbiology at Wageningen University and stored at −80°C before being processed. A total number of 739 fecal samples were collected at these five timepoints. Participants with at least one fecal sample at these assessment moments were included in the present study (*N* = 187).

Behavioral measures were collected with questionnaires at 6 and 10 years of age. Additionally, we recorded the following variables as extrinsic factors that may predict variance in the gut microbiota: child age, child sex, birth weight, delivery mode, frequency of breastfeeding and formula intake in the first 27 weeks of life (mothers were required to weekly record the average number of breastfeeding and formula intake per day), the age of first solid food introduction, and use of antibiotics in the first 10 years of life.^80,81^ Finally, we also measured dietary intake at age 10 by a food frequency questionnaire.

### Measures

#### Gut microbiota composition

In brief, DNA extraction was performed using the Maxwell 16 Total RNA system (Promega, Wisconsin, USA) with 0.01–0.13 g of fecal sample and Stool Transport and Recovery Buffer (STAR; Roche Diagnostics Corporation, Indianapolis, IN), as reported previously.^82^ Amplification was performed on the V4 region of 16S ribosomal RNA (rRNA) gene in duplicate, generating amplicons with a length of around 290 bp.^82^ Each PCR reaction comprised of 10 µl of 5x Phusion Green HF Buffer (Thermo Scientific, US), 1 µl of 10 µM barcoded primers 515 F-n(5’-GTGYCAGCMGCCGCGGTAA-3’) and 806 R-n(5’- GGACTACNVGGGTWTCTAAT-3’),^83,84^ 1 µl of 10 mM dNTPs mix (Promega Corporation, US), 0.5 µl of 2 U/µl Phusion Green Hot Start II High-Fidelity DNA polymerase (Thermo Scientific, US), 36.5 µl of Nuclease-free water and 1 µl of 20 ng/µl DNA template. PCR was carried out as previously described,^82^ with modification: initial denaturation (98°C, 30 s), 25 cycles of denaturation (98°C, 10 s), annealing (50°C, 10 s), extension (72°C, 10s), and elongation (72°C, 7 min). The presence and length of PCR products was then verified by gel electrophoresis. PCR products were purified by the HighPrep® PCR kit (MagBio Genomics, Alphen aan den Rijn, Netherlands), according to the instructions of the kit. DNA concentration of purified samples was measured using a fluorometer (DS-11; DeNovix) with Qubit® dsDNA BR Assay Kit (Life Technologies, Leusden, Netherlands). Two hundred nanograms of barcoded samples was pooled in libraries comprising 69 uniquely tagged samples, 2 of which were artificial control communities representative of human gut microbiota.^85^ The mixture was purified again by HighPrep® PCR kit to a final volume of 40 µl.

16S rRNA gene sequencing was completed on the Illumina sequencing platform at Eurofins Genomics, Germany. *NG-Tax* was used for processing of 16S rRNA gene sequence data.^85,86^ Only reads with matching barcodes were kept. Subsequently, amplicon sequence variants (ASVs) were identified on a per sample basis. Taxonomic assignment of ASVs was performed referring to SILVA_132_SSU 16S rRNA gene reference database.^87^ A total of 113,413,327 reads were obtained from the sequencing. Basic descriptions of reads, ASVs and genera numbers were displayed in Table S16.

#### Behavioral measures

##### CBCL

The Child Behavior Checklist 4–18 (CBCL) is a 118-item questionnaire, assessing problem behaviors of children from ages 4 to 18, scored on a 3-point scale.^88^ The CBCL includes internalizing and externalizing subscales. Higher scores indicate more behavioral problems. Mothers were required to complete the CBCL when their children were 6 years old.

##### SDQ

The Strengths and Difficulties Questionnaire (SDQ) is a 25-item scale, evaluating problem behaviors in children from ages 4 to 16, and scored on a 3-point scale (Table S17).^89^ The SDQ includes internalizing and externalizing subscales. Higher scores represent more behavioral problems. Although the SDQ is shorter than the CBCL, it has verified equivalent ability to assess problem behaviors.^90^ Due to practical reasons, children were asked to complete the SDQ rather than the CBCL when they were 10 years old. Mothers also completed the SDQ when their children were 10. Considering known discrepancies between mothers and children in assessing problem behaviors at this age,^91^ we included both maternal and child reports in the current study.

##### Questionnaire reliability

To check the internal consistency of the questionnaires, we calculated ω_total_ estimates by using the R package *psych*.^92,93^ Given ω_total_ estimates were incalculable for the CBCL, we computed Cronbach’s α values for this questionnaire instead. The resulting internal consistency estimates were as follows: the maternal CBCL, α_internalizing _= 0.82, α_externalizing_ = 0.84; the maternal SDQ, ω_total-internalizing _= 0.72, ω_total-externalizing _= 0.80; the child SDQ, ω_total-internalizing _= 0.63, ω_total-externalizing _= 0.59. Hence, most estimates indicated acceptable or good internal consistency of the subscales. The estimates of the child SDQ were considered questionable, but in line with earlier Dutch studies, and thus used in the analyses.^94^

#### Extrinsic factors

Extrinsic factors included (1) Child age when stool samples were collected; (2) Delivery mode (i.e., assisted vaginal delivery, non-assisted vaginal delivery and C-section); (3) Birth weight; (4) Breastfeeding (for samples collected at age 1, 3 and 4 months, breastfeeding = average number of daily breastfeedings with respect to total number of daily milk feedings (in percentage) until stool collection day; for samples collected at age 6 and 10 years, breastfeeding = average number of daily breastfeedings with respect to total number of daily milk feedings (in percentage) in the first 27 weeks.); (5) Child age when solid food was first introduced; (6) Child sex (female or male); (7) Total number of antibiotic treatments from birth to stool collection day (infants and children); (8) Whether a child at age 6 or 10 took antibiotics in the past 1 year (yes or no).

### Statistical analyses

All analyses were performed in R (version 3.6.1).^95^

#### First aim: Gut microbiota development in the first 10 years of life

##### Gut bacterial clusters and transition patterns

To investigate normative development of the gut microbiota, we identified gut bacterial clusters based on their compositional features at the genus level by Dirichlet multinomial mixtures (DMM) models, known for their superior advantage of handling sparse data.^96^ Considering the reproducibility and stability of the optimal clusters, we split the samples into two parts, infancy (i.e., 1, 3 and 4 months) and childhood (6 and 10 years), and performed separate DMM models afterward. The optimal number of clusters was determined by lowest Laplace approximation scores. As the combination of clusters varied between runnings, we repeated DMM models multiple times and then selected the combinations that appeared the most frequently (Tables S18 and S19).

##### Characteristics of gut bacterial clusters

The relative abundances of the top 15 predominant genera in infancy and childhood were displayed in heatmaps by the *ComplexHeatmap* package.^97^ Phylogenetic alpha diversity was computed by using the *picante* package^98^ and compared between bacterial clusters by Wilcoxon rank sum tests. Obtained *p* values from the comparisons were then adjusted by FDR. Beta diversity was compared between bacterial clusters by using unweighted or weighted Unifrac distance of genera relative abundances via the *vegan* package.^99^

Additionally, the functional potential of the microbial community was predicted by Picrust2 (phylogenetic investigation of communities by reconstruction of unobserved states) approach.^47,48^ Predicted gene family counts (i.e., Kyoto Encyclopedia of Genes and Genomes (KEGG) orthologs and MetaCyc pathways) for each sample were transferred into relative abundance data. Kruskal–Wallis tests for multiple-group comparisons were first performed on relative abundances of predicted functions between bacterial clusters in infancy and childhood, respectively. The predicted function, with an FDR-corrected *p* value less than 0.05 and the average relative abundance more than 0.5%, was further compared between every two bacterial clusters by Wilcoxon rank sum tests.

##### Effects of extrinsic factors

First, Redundancy Analysis (RDA) was used to measure simple effects of extrinsic factors on the gut microbiota for each of five ages (i.e., 1, 3 and 4 months, and 6 and 10 years), and then infancy (i.e., 1, 3 and 4 months) and childhood (i.e., 6 and 10 years). Child age, delivery mode, birth weight, breastfeeding and child sex were considered in infancy. Because only two infants took antibiotics in the first 4 months of life, we did not consider this factor in infancy RDA models. Similarly, as only one child started consuming solids before the stool collection at 4 months, this factor was not considered in infancy RDA models. As for samples in childhood, all extrinsic factors mentioned before were included. Quantitative extrinsic factors were converted to z-scores before use. Second, RDA was performed to measure conditional effects for extrinsic factors with significant simple effects. To avoid potential strong multicollinearity in RDA when assessing conditional effects, we required variance inflation factors (VIFs) of all extrinsic factors to be less than three.^100^ Third, RDA tri-plots were drawn by the *ggplot* package.^101^ All RDA models were built based on Bray-Curtis dissimilarity matrices calculated from log-transformed relative abundances at the genus level, via the *vegan* package.^99^ Permutation tests with 1000 permutations were used to determine the significance of variance explained by extrinsic factors.

#### Second aim: Associations of the gut microbiota with internalizing and externalizing behavior in middle childhood

##### Behavioral differences between bacterial clusters

To compare behavioral measures (i.e., internalizing and externalizing behavior) between bacterial clusters, we conducted Wilcoxon rank sum tests with FDR adjustment.

##### RDA models

RDA models were established to determine how much variance in microbial composition could be explained by behavioral measures (i.e., internalizing and externalizing behavior) with and without accounting for extrinsic factors with significant conditional effects. Internalizing and externalizing behavior scores were standardized to *z*-scores before use. Then, RDA tri-plots were drawn by the *ggplot* package.^101^ The same VIF requirements, matrices type and permutation tests as for extrinsic factors were also adopted here.

##### PRC models

Principal Response Curves (PRC) analysis is a method that enables contrasting time series of experimental groups with a time series of a reference group.^102^ Therefore, it was used to select the genera with relatively large differences in relative abundance across ages between behavior groups. Two behavior groups were set for each of the behavior types (internalizing and externalizing behavior). The experimental group (H) included the individuals with behavior scores in the top quartile, while the reference group (L + M) consisted of all remaining individuals. Relative abundance data were preprocessed with log-transformation. PRC models were generated using the *vegan* package.^99^ PRC diagrams were visualized by the *ggplot* package.^101^ The first principal component of the variance explained by behavior groups in time, called canonical coefficient, was displayed on the y-axis. The age points were shown on the x-axis. Another vertical axis, named taxon weight, was drawn to elucidate the affinity of the different genera with the response. Wilcoxon rank sum tests with FDR adjustment were further applied for comparing the differences of selected genera from PRC models between behavior groups at individual time points.

##### MLM models

Multilevel modelling (MLM) models were conducted to measure the associations of internalizing and externalizing behavior with diversity and selected genera when accounting for extrinsic factors. Selected genera included bacteria with absolute values of taxon weights more than 0.6 and prevalence above 0.2. For MLM models including samples in infancy, extrinsic factors consisted of age, breastfeeding, delivery mode, birth weight and gender. For MLM models in childhood, these factors, as well as antibiotics and age when solid food was introduced, were included. Quantitative extrinsic factors, behavior scores and diversity were converted to *z*-scores before use. Log transformation was applied to relative abundance data at the genus level. To avoid strong multicollinearity, generalized variance inflation factors (GVIFs) were required to be less than 3. MLM models were built by the *lmerTest* package.^103^

#### Significance

Statistically significant level was required with *p* value less than 0.05.

## Supplementary Material

Supplemental Material

## Data Availability

Due to the BIBO datasets are parts of an ongoing longitudinal study, the data that support the findings of this study cannot be made publicly available but are available upon request from C de Weerth (Carolina.deWeerth@radboudumc.nl; https://doi.org/10.5281/zenodo.5500644).
