# Supplementary material for: Development of the gut microbiota in healthy children in the first ten years of life: associations with internalizing and externalizing behavior
Source: Gut Microbes. 2022 Feb 19;14(1):2038853. doi: 10.1080/19490976.2022.2038853 (PMC8865293; doi:10.1080/19490976.2022.2038853)
Supplement: Supplemental Material [file KGMI_A_2038853_SM3188.zip › supplementary/GM_BP_SupplementaryFigures_V2.docx]

Figure S1. Box plots indicating phylogenetic diversity in the different bacterial clusters. Diversity indices were calculated based on amplicon sequence variants in each bacterial cluster. The boxes range from 25th to 75th percentiles, with center lines indicating medians. Outliers are displayed as points. Asterisks indicate *p* values < 0.05 (with FDR correction).

Figure S2. Beta diversity between bacterial clusters. (a-b) Beta diversity was calculated from unweighted Unifrac distance obtained from relative abundance data at the genus level. (c-d) Beta diversity was computed from weighted Unifrac distance obtained from genera relative abundance data. Adonis reflects the significance of comparisons (N=1000 permutations). Betadisper refers to the significance of the homogeneity of variances of which a value higher than 0.05 means variances are homogeneous (N=1000 permutations).

Figure S3. Differences of internalizing behavior between bacterial clusters. CBCL_M_Int_6y: internalizing behavior measured by maternal CBCL at age six. SDQ_M_Int_10y: internalizing behavior measured by maternal SDQ at age ten. SDQ_C_Int_10y: internalizing behavior measured by child SDQ at age ten. Wilcoxon rank sum tests were conducted with FDR adjustment. No significant differences were observed in internalizing behavior between the clusters.

Figure S4. Differences of externalizing behavior between bacterial clusters. CBCL_M_Ext_6y: externalizing behavior measured by maternal CBCL at age six. SDQ_M_Ext_10y: externalizing behavior measured by maternal SDQ at age ten. SDQ_C_Ext_10y: externalizing behavior measured by child SDQ at age ten. Wilcoxon rank sum tests were conducted with FDR adjustment. No significant differences were observed in externalizing behavior between the clusters.
